# Preconception air pollution exposure and glucose tolerance in healthy pregnant women in a middle-income country

**DOI:** 10.1186/s12940-020-00682-y

**Published:** 2020-12-09

**Authors:** Moslem Lari Najafi, Mehdi Zarei, Ali Gohari, Leyla Haghighi, Hafez Heydari, Mohammad Miri

**Affiliations:** 1 Pharmaceutical Sciences and Cosmetic Products Research Center, Kerman University of Medical Sciences, Kerman, Iran; 2 Department of Physical Education and Sport Science, Faculty of Human Science, University of Neyshabur, Neyshabur, Iran; 3 Cellular and Molecular Research Center, Sabzevar University of Medical Sciences, Sabzevar, Iran; 4 Non-Communicable Diseases Research Center, Department of Environmental Health, School of Health, Sabzevar University of Medical Sciences, PO Box 319, Sabzevar, Iran

**Keywords:** Air pollution, Glucose tolerance, Pregnancy, Traffic

## Abstract

**Background:**

Preconception exposure to air pollution has been associated with glucose tolerance during pregnancy. However, the evidence in low and middle-income countries (LMICs) is under debate yet. Therefore, this study aimed to assess the relationship between exposure to ambient particulate matter (PM) and traffic indicators with glucose tolerance in healthy pregnant women in Sabzevar, Iran (2019).

**Methods:**

Two-hundred and fifty healthy pregnant women with singleton pregnancies and 24–26 weeks of gestations participated in our study. Land use regression (LUR) models were applied to estimate the annual mean of PM_1_, PM_2.5_ and PM_10_ at the residential address. Traffic indicators, including proximity of women to major roads as well as total streets length in 100, 300 and 500 m buffers around the home were calculated using the street map of Sabzevar. The oral glucose tolerance test (OGTT) was used to assess glucose tolerance during pregnancy. Multiple linear regression adjusted for relevant covariates was used to estimate the association of fasting blood glucose (FBG), 1-h and 2-h post-load glucose with PMs and traffic indicators.

**Results:**

Exposure to PM_1_, PM_2.5_ and PM_10_ was significantly associated with higher FBG concentration. Higher total streets length in a 100 m buffer was associated with higher FBG and 1-h glucose concentrations. An interquartile range (IQR) increase in proximity to major roads was associated with a decrease of − 3.29 mg/dL (95% confidence interval (CI): − 4.35, − 2.23, *P*-value < 0.01) in FBG level and − 3.65 mg/dL (95% CI, − 7.01, − 0.28, *P-*value = 0.03) decrease in 1-h post-load glucose.

**Conclusion:**

We found that higher preconception exposure to air pollution was associated with higher FBG and 1-h glucose concentrations during pregnancy.

**Supplementary Information:**

The online version contains supplementary material available at 10.1186/s12940-020-00682-y.

## Introduction

Gestational diabetes mellitus (GDM) has been associated with pregnancy complications, including macrosomia, hypertension, preeclampsia and premature birth and stillbirth [1]. In recent decades, the GDM prevalence has increased significantly [2]; however, about half of them have no classic risk factors [3, 4]. The available evidence suggested that environmental pollutants, e.g., air pollution, could act as a risk factor in glucose tolerance and glucose homeostasis in healthy women [5–15]. However, the results of these studies are inconsistent. Some of these studies reported a significant relationship between exposure to particulate matter (PM) with impaired glucose tolerance (IGT) [7, 16, 17] and increased risk of GDM [8, 18, 19]; however, other evidence reported opposing results [9, 12, 16]. These studies used different diagnostic criteria and had limitations in the timing of GDM development [18]. Moreover, only three studies investigated the preconception window [9, 18, 19]. In an effort to clarify the potential mechanisms, the available evidence has reported higher exposure to air pollution is associated with elevated blood glucose levels, a potential sign of increased insulin resistance, GDM and type 2 diabetes development in later life [20–23].

The emerging evidence indicated that the pre-pregnancy period might be a crucial time-window and higher exposure to pollutions could lead to glucose intolerance and GDM development [24]. So far, limited studies have been investigated the relationship between exposure to traffic indicators and PMs with glucose tolerance in healthy pregnant women [16, 17]. Moreover, the available studies on the association of preconception exposure to traffic indicators and PMs with glucose concentrations obtained through oral glucose tolerance test (OGTT) have been exclusively conducted in developed countries. However, the available evidence in low and middle-income countries (LMICs) with more rapid urbanization is still scarce. Given the variation in the observed associations between air pollution exposure and OGTT in different settings [9, 18, 19], it is not clear to what extent the results from developed countries could be generalized to LMICs. Therefore, this study aimed to assess the relationship between preconception exposure to traffic indicators as well as PMs at mother residence and OGTT results, a marker of glucose intolerance in healthy pregnant women in a middle income country (i.e., Iran).

## Material and methods

### Study area

**Fig. 1:**
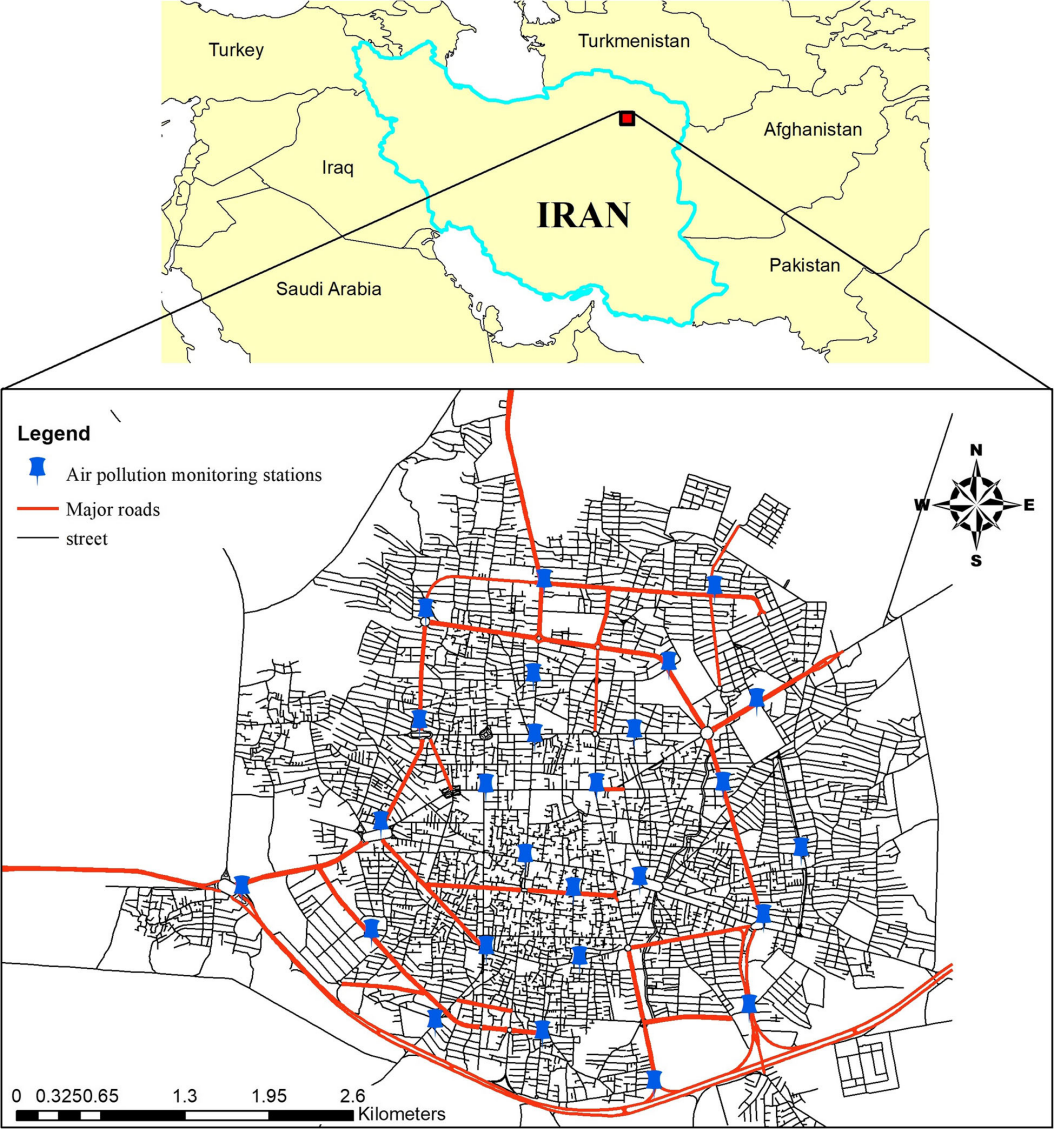
Study area, air pollution monitoring stations and major roads

This cross-sectional study was conducted in Sabzevar (coordinates: 36°12′ N 57°35′, elevation: 977.6 m) a town in Khorasan Razavi province, Iran. Sabzevar is a city with an arid climate and annual average rainfall lower than 180 mm, the annual average temperature of 16 °C and relative humidity of 43%. Based on the last census in 2016, the population of Sabzevar is about 240,000 [25]. Figure 1 showed the air pollution monitoring stations, street map and major roads of Sabzevar.

**Fig. 2:**
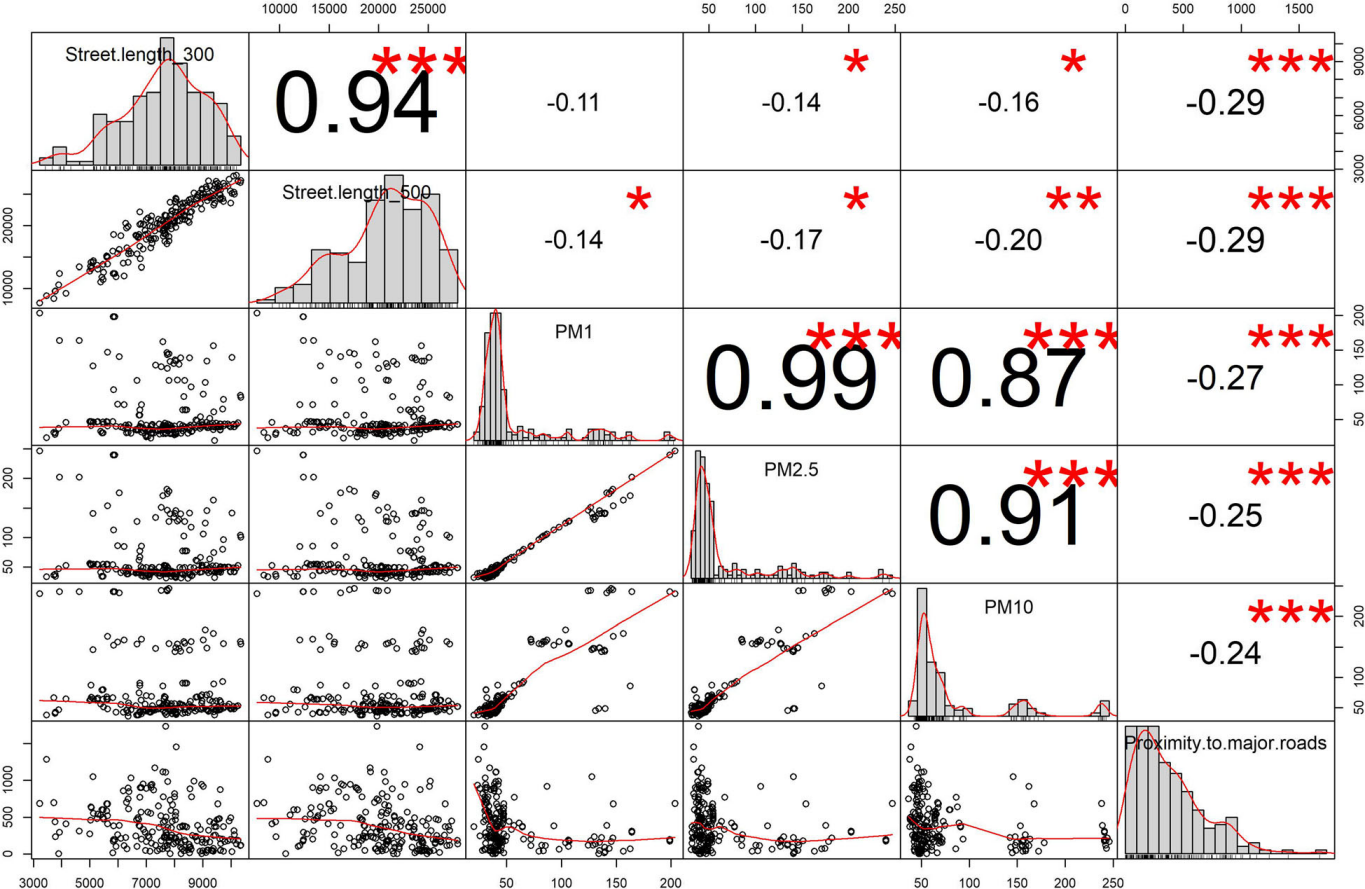
Spearman correlation between PMs and traffic indicators (* significant level of 0.1, ** significant level of 0.05 and *** significant level of 0.01)

### Population setting

The pregnant women how were recruited to only Sabzevar Health Center for GDM screening during Jun 2019 to September 2019 were invited to this study. The inclusion criteria were including the gestational age of 24 to 26 weeks at enrollment, lived in Sabzevar during and before the pregnancy (at least 1 year) and singleton pregnancy. The exclusion criteria were including mothers who had GDM (because the glucose metabolism and adipokine concentrations in the women with GDM is different compare to healthy women; moreover, there is insulin resistance and higher oxidative stress in these women that can affect glucose regulation in the women with GDM), preeclampsia, hypertension, change their residence during pregnancy and work outside of the home. Moreover, the mothers how were active smokers during and before pregnancy were excluded from our study. It should be note that, there is not any variation in race or ethnicity of the inhabitants of Sabzevar (all inhabitants are Persians). From more than 5000 pregnant women who referred to the only Sabzevar Health Center for GDM screening, 250 of them had eligible criteria and applied to join in this study. Prior to entering the study, the inclusion/exclusion criteria, research aims and procedures were described to all pregnant women and all participants signed the consent form approved by the Ethics Committee of Sabzevar University of Medical Sciences (IR.MEDSAB.REC.1397.012). Socioeconomic information and lifestyle factors were obtained using a prepared questionnaire by face-to-face interviews.

### Exposure assessment

#### Ambient particulate matter

The developed land use regression (LUR) models for Sabzevar were applied to estimate the preconception exposure to ambient PMs (i.e., PM_1_, PM_2.5_, and PM_10_) at the residential address. The PMs data were estimated based on annual mean concentrations before pregnancy. The details of developed models have been described in detail elsewhere [26]. Briefly, the PM_1_, PM_2.5_, and PM_10_ concentrations were measured using 26 air pollution monitoring stations installed in the different microenvironments. A mobile monitoring device (HAZ-DUST EPAM 5000, USA) was used to measure the PMs pollutants based on the method described by the United States Environmental Protection Agency (USEPA). The LUR models were generated based on the annual average of PMs concentrations. A step-forward algorithm was used to develop LUR models. The main important variables which applied in developing LUR models were including urban morphology, population density, ten different land use, traffic and geographic location of monitoring stations. The generated models were able to predict 68, 72 and 75% of the variation of annual PM_1_, PM_2.5_ and PM_10_ concentrations in Sabzevar. The LUR models performance was evaluated using the leave-one-out cross-validation (LOOCV) method. More details of LUR models validation are presented in Table S1 of Supplemental Materials.

#### Traffic density indicators

Previous studies introduced street length and proximity to major roads as traffic indicators [27, 28]. Therefore we used total streets length in 100, 300 and 500 m buffer around the mother residence and proximity of women to major roads as indicators of exposure to traffic. These indicators were calculated using Sabzevar street map, provided by Sabzevar municipality in ArcGIS v 10.5 software.

### Glycemic status screening

During the study period, GDM screening in Iran was based on OGTT results. Glycemic status was assessed using 2-h, 75-g OGTT [29]. The OGTT was performed in the morning (8:00–9:00 AM) in the outpatient clinic, and the participants fasted for at least ten h prior to the tests. Normal glucose tolerance was determined according to the American Diabetes Association (ADA) (2008) criteria [30]. Subjects with FBG ≥ 95 mg/dL at baseline, ≥ 180 mg/dL at 1-h, ≥ 155 mg/dL at 2-h were considered as normal glucose tolerance (NGT). The OGTT considered abnormal if one or two glucose concentrations exceeded mentioned concentrations. The pregnant women with 1-h glucose concentration higher than cutoff and normal FBG concentration were classified as impaired glucose tolerance that have different outcome of glucose homeostasis [31]. Therefore, we excluded mothers who had one higher glucose concentration than NGT.

#### Glucose concentration measurement

The glucose oxidase method (Pars Azmoon, Tehran, Iran) and autoanalyzer (BT-3000) were applied to measure the venous serum glucose concentration of mothers in the reference lab of Sabzevar Health Center according to a standard clinical protocol [32]. The accuracy and precision of the assay were controlled by positive and negative controls as well as standard glucose concentration, daily.

### Statistical analysis

#### Main analysis

The distribution of data was tested using the Shapiro-Wilk test. We developed linear regression models (MLR) to estimate the change in the FBG, 1-h and 2-h glucose concentrations associated with an interquartile range (IQR) increase in traffic indicators and PMs exposure (one at a time). The MLR models were further adjusted for a *prior* potential covariates including maternal age (year, continuous), tobacco smoke exposure at home (yes/ no) [33] and two indicators of neighborhood socioeconomic status including percentages of unemployment and illiterate adults per census tract (based on the 2016 census). All statistical analysis was performed using Stata version 15 (Stata Corp LP, College Station, Texas).

## Results

About 5% (250 participants) of women that attended the Sabzevar Health Center for GDM screening participated in our study. The statistical summary of the participants, their PMs exposure as well as traffic indicators and FBG, 1-h and 2-h glucose concentrations are presented in Table 1. The median (IQR) age of pregnant women was 28 (8) years. The median (IQR) of pre-pregnancy BMI was 21.2 (5.9) kg/m^2^. The median (IQR) of FBG, 1-h and 2-h glucose concentrations were 69 (8), 112 (35), and 100 (26), mg/dL, respectively. The median (IQR) of proximity to major roads and total street length in 100, 300 and 500 m buffers were 321 (388), 905 (257), 7756 (2035) and 20,704 (6292) meters, respectively. Moreover, median (IQR) of estimated PM_1_, PM_2.5_ and PM_10_ at residential address were 40.8 (14.7), 47.4 (21.5) and 52.9 (23.7) μg/m^3^, respectively (Table 1).

**Table 1:** Descriptive statistics of pregnant women, PMs and traffic indicators

Variables	In study year
**Pregnant Women characteristics**	
Age (year); median (IQR)	28 (8)
Pre-pregnancy BMI (kg/m^2^); median (IQR)	21.2 (5.9)
Gestational age (week); median (IQR)	26 (4)
Self-reported tobacco exposure at home	
Yes; N (%)	75 (30)
No; N (%)	175 (70)
Abortion history	
Yes; N (%)	90 (25)
No; N (%)	160 (75)
Family history of diabetes	
Yes; N (%)	24 (11)
No; N (%)	189 (89)
Parity (N); median (IQR)	2 (2)
Illiterate adults per census tract (%); median (IQR)	22.2 (15.3)
Unemployed adults per census tract(%); median (IQR)	7.0 (4.5)
**Glucose concentrations (mg/dL); median (IQR)**	
FBG	69 (8)
1-h post-load glucose	112 (35)
2-h post-load glucose	100 (26)
**Particulate matter pollutants (μg/m**^**3**^**); median (IQR)**	
PM_1_	40.8 (14.7)
PM_2.5_	47.4 (21.5)
PM_10_	52.9 (23.7)
**Traffic indicators (m); median (IQR)**	
Total street length in a 100 m buffer	905 (257)
Total street length in a 300 m buffer	7756 (2035)
Total street length in a 500 m buffer	20,704 (6292)
Proximity to major roads	321 (388)

*IQR* interquartile range, *BMI* body mass index, *FGB* fasting blood glucose

Spearman correlation of PM_1_, PM_2.5,_ PM_10_, proximity to major roads and total street length in different buffers at the residential address of pregnant women are shown in Fig. 2. Moreover, correlation coefficients of traffic indicators and PMs with FBG, 1-h and 2-h post-load glucose are shown in Fig. S1 of Supplemental Materials. There was a negative correlation between proximity to major roads and PM_1_, PM_2.5_ and PM_10_ (r = − 0.27, − 0.25 and − 0.24, respectively). Furthermore, we observed a strong correlation between estimated PMs at the residential address. A moderate positive correlation was observed between estimated PMs and total street length in 100 m buffer (r ranged from 0.18 to 0.32).

The results of the associations of exposure to traffic indicators and PMs with FBG, 1-h and 2-h post-load glucose concentrations in healthy pregnant women are presented in Table 2. Overall, higher exposure to ambient PM_1_, PM_2.5_ and PM_10_ were associated with higher FBG. Moreover, higher TSL-100 m was associated with higher FBG and 1-h. post-load glucose concentrations. Higher proximity to major roads was negatively associated with FBG and 1-h. post-load glucose concentrations (Table 2).

**Table 2:** The regression coefficient of exposure to air pollution with FBG, 1-h and 2-h post-load glucose concentrations in healthy pregnant women

Exposure	Outcome		β-coefficient (95% CI)	***P***_value
**PM pollutants**				
PM_1_	FBG	Crude	0.80 (0.48, 1.11)	< 0.01
		Adjusted^a^	0.69 (0.38, 1.00)	< 0.01
	1-h post-load glucose	Crude	0.54 (−0.44, 1.53)	0.27
		Adjusted	0.41 (−0.56, 1.37)	0.41
	2-h post-load glucose	Crude	0.55 (− 0.29, 1.39)	0.19
		Adjusted	2.86 (−2.14, 7.86)	0.26
PM_2.5_	FBG	Crude	0.71 (0.38, 1.00)	< 0.01
		Adjusted	0.61 (0.29, 0.93)	< 0.01
	1-h post-load glucose	Crude	0.42 (−0.58, 1.4)	0.40
		Adjusted	0.34 (−0.65, 1.33)	0.50
	2-h post-load glucose	Crude	0.41 (−0.45, 1.28)	0.34
		Adjusted	3.13 (−1.98, 8.23)	0.23
PM_10_	FBG	Crude	0.22 (0.04, 0.40)	< 0.01
		Adjusted	0.19 (0.01, 0.37)	0.04
	1-h post-load glucose	Crude	0.11 (−0.43, 0.66)	0.68
		Adjusted	0.14 (−0.39, 0.67)	0.60
	2-h post-load glucose	Crude	0.14 (−0.32, 0.60)	0.55
		Adjusted	2.45 (−0.27, 5.17)	0.08
**Traffic indicators**				
Street length in a 100 m buffer	FBG	Crude	2.70 (2.13, 3.27)	< 0.01
		Adjusted	2.57 (1.99, 3.19)	< 0.01
	1-h post-load glucose	Crude	3.46 (1.51, 5.41)	< 0.01
		Adjusted	3.44 (1.49, 5.39)	< 0.01
	2-h post-load glucose	Crude	2.18 (0.49, 3.87)	0.01
		Adjusted	−2.95 (−13.36, 7.46)	0.58
Street length in a 300 m buffer	FBG	Crude	0.22 (− 1.17, 1.63)	0.75
		Adjusted	−0.16 (−1.53, 1.22)	0.82
	1-h post-load glucose	Crude	0.00 (−4.15, 4.16)	0.99
		Adjusted	−1.00 (−5.05, 3.06)	0.63
	2-h post-load glucose	Crude	−0.36 (−3.92, 3.20)	0.84
		Adjusted	−2.46 (−23.48, 18.57)	0.82
Street length in a 500 m buffer	FBG	Crude	0.07 (−1.37, 1.52)	0.92
		Adjusted	−0.29 (−1.71, 1.13)	0.69
	1-h post-load glucose	Crude	0.20 (−4.08, 4.50)	0.92
		Adjusted	−0.84 (−5.04, 3.77)	0.70
	2-h post-load glucose	Crude	−0.55 (−4.22, 3.12)	0.76
		Adjusted	−5.89 (−27.66, 15.87)	0.59
Proximity to major roads	FBG	Crude	−3.49 (−4.56, −2.43)	< 0.01
		Adjusted	−3.29 (−4.35, −2.23)	< 0.01
	1-h post-load glucose	Crude	−4.17 (−7.58, −0.75)	< 0.01
		Adjusted	−3.65 (−7.01, −0.28)	0.03
	2-h post-load glucose	Crude	−3.74 (−6.66, −0.82)	0.01
		Adjusted	−4.39 (−22.00, 13.23)	0.62

^a^Adjusted for the maternal age, exposure to environmental tobacco smoke, percentage of illiterate as well as unemployed adults per census tract

*CI* confidence interval, *FBS* fasting blood glucose. The regression coefficients were reported based on 1 IQR increase in PM_1_, PM_2.5_, PM_10_, total street length in different buffers and proximity to major roads

In fully adjusted models, a one-IQR increase in concentration of PM_1_, PM_2.5_ and PM_10_ was associated with increase of 0.69 mg/dL (95% confidence interval (CI): 0.38, 1.00, *P*-value < 0.01), 0.61 mg/dL (95% CI: 0.29, 0.93, *P-*value < 0.01) and 0.19 mg/dL (95% CI: 0.01, 0.37, *P-*value = 0.04) in FGB concentration. Furthermore, one-IQR increase in total street length was associated with an increase of 2.57 mg/dL (95% CI: 1.99, 3.19, *P-*value < 0.01) in FBG concentration. There was also a significant negative association between proximity to major roads and FBG concentration (β = − 3.29, 95% CI: − 4.35, − 2.23, *P*-value < 0.01). We did not find any significant association for total street length in 300 and 500 m buffers and FBG.

Higher total street length in 100 m buffer was associated with higher 1-h post-load glucose concentration. In fully adjusted model, a one-IQR increase in total street length in 100 m buffer was associated with an increase of 3.44 mg/dL (95% CI: 1.49, 5.39, *P*-value < 0.01) in 1-h post-load glucose concentration. Moreover, a one-IQR increase in proximity to major roads was associated with a decrease of − 3.65 mg/dL (95% CI: − 7.01, − 0.28, *P-*value =0.03) in 1-h post-load glucose concentrations. The associations of total street length in 300 and 500 m buffers, as well as PM_1_, PM_2.5_ and PM_10_, were not statistically significant.

In this study, we did not observe any significant association between traffic indicators as well as PMs exposures and 2-h post-load glucose concentration (Table 2).

## Discussion

To the best of our knowledge, this study is one of the first to evaluate the association of preconception exposure to traffic indicators and air pollution with the glucose concentration obtained in OGTT of healthy pregnant women in a middle-income country. The main advantage of our study is the use OGTT results as a sensitive test for glucose homeostasis evaluations. We found that exposure to PM_1_, PM_2.5_ and PM_10_ was positively associated with FBG concentration, demonstrating that the levels of these pollutants might increase the risk of glucose intolerance. Moreover, the total street length in 100 m buffer was positively associated with FBG and 1-h post-load glucose concentrations. Furthermore, proximity to major roads was negatively associated with FBG and 1-h post-load glucose concentrations.

In our study, the mean of estimated PM_1_, PM_2.5_ and PM_10_ at the residential address were 56.5, 65.0 and 76.6 μg/m^3^, respectively. There is not any guideline/standard for ambient PM_1_ concentration; however, the estimated concentrations of PM_2.5_ and PM_10_ were 6.5 and 3.8 times higher than WHO guidelines (based on annual mean, 10 μg/m^3^ for PM_2.5_ and 20 μg/m^3^ for PM_10_) [34]. The PMs concentrations reported in our study were comparable with previous studies in Sabzevar [35, 36] and other cities of Iran, e.g., among residents Hamadan with annual exposure of 41 μg/m^3^ for PM_2.5_ and 68 μg/m^3^ for PM_10_ [37] and Mashhad with annual exposure of 40.7 μg/m^3^ for PM_2.5_ and 82.9 μg/m^3^ for PM_10_ [38].

I this study, increase in concentration of PM_1_, PM_2.5_ and PM_10_ was associated with increase of 0.69, 0.61 and 0.19 mg/dL in FGB concentration. Given that this is the first studies looking at the association between exposure to PM_1_, PM_10_ and traffic indicators with FBG, 1-h and 2-h post-load glucose concentrations obtained in OGTT as indicators of glucose homeostasis in healthy pregnant women; we could not compare our finding regarding these pollutants with results of previous studies. However, a study by Lu et al. 2017 on the 3859 subjects aged over 30 years found that higher FBG, 1-h, 2-h and 3-h glucose concentrations in pregnant women who lived in areas with higher PM_2.5_ level [17]. A part of this study (i.e., significant positive association between PM_2.5_ exposures and FBG concentration) is in line with our findings; while, the associations of 1-h and 2-h glucose were inconsistent with our findings. Genetic difference [39], different lifestyle (e.g., physical activity) [40] and diet [41] in our participants compared to papulation who studied by *Lu* et al.*,* could explain this inconsistently. Another study by Fleisch et al. 2014 on 2093 women found that second-trimester PM_2.5_ exposure was associated with IGT occurrence but not GDM [16]. Choe et al. 2019 reported that PM_2.5_ exposure in 2nd trimester was associated with GDM development [42]. A population-based retrospective cohort study by Shen et al. 2017, found higher PM_2.5_ exposure during 12 week preconception period as well as first two trimesters of pregnancy was significantly associated with increase in the risk of GDM in pregnant women [19]. Moreover, the relationship between air pollution exposures and glucose homeostasis in non-pregnant healthy adults has been reported in previous studies. Peng et al. 2016 found that PM_2.5_ exposure was significantly associated with increase in FBG concentration in non-diabetic subjects [43]. A study by Riant et al. 2018 on 2895 participants aged 40–65 years in France reported that PM_10_ exposure was associated with higher FBG and HbA1c [44]. A study by Chen et al. 2016, reported that short-term exposure to PM_10_ (4 days) was associated with higher FBG concentration as well as IFG occurrence [45]. A systematic review and meta-analysis by Elshahidi et al. 2019 found that higher PM_2.5_ and PM_10_ exposures were associated with GDM development [46]. These reports are in line with our findings. In the other hand, many previous systematic review and meta-analysis as well as cohort studies reported that higher FBG levels could increase the risk of GDM and type 2 diabetes in later life [47–51]. Therefore, higher preconception exposure to PMs might increase the risk of type 2 diabetes in these participants in later life.

We found, FBG and 1-h post-load glucose concentrations were positively associated with total street length in 100 m buffer and negatively associated with proximity to major roads. There is limited evidence that investigated the relationship between traffic-related air and noise pollution with glucose tolerance during pregnancy [31, 52, 53]. Hooven et al. 2009 investigated the association between residential proximity to traffic and outcome of glucose homeostasis during pregnancy and reported there was no significant association between traffic indicators and GDM occurrence [52]. Pedersen et al. 2017 examine the association of exposure to air and noise pollution in pregnant women and reported that there was no significant association of exposure to both pollutants and GDM development [31]. In contrast, Malmqvist et al. 2013 in a study based on birth registry data of 81,000 pregnant women in Sweden, found that exposure to traffic indicators was significantly associated with GDM development [53]. In our study, we found a positive correlation between total street length in 100 m buffer with PMs as well as a negative correlation between proximity to major roads and PMs concentrations. Previous studies have shown that higher street length was significantly correlated with higher levels of PMs, especially in the smaller buffer sizes (e.g., 100 m) [25, 27, 35, 36]. These results could be explained our findings on the significant association of traffic indicators and glucose intolerance.

Although the precise mechanisms of the effect of traffic indicators and air pollution exposure on glucose tolerance are not fully understood, a number of potential mechanisms have been proposed. It has been shown that inhaled PMs into respiratory tract can pass through the alveolar cell and affect metabolism in extrapulmonary organs, e.g., liver [36, 54]. Similarly, non-water-soluble PMs with an aerodynamic diameter of ≤0.1 μm could alter glucose metabolism by entering the target cells [25, 55]. Another potential mechanism could be inhaled PMs that activate immunity cells, resulting in cytokines release [56, 57]. Some of these cytokines change glucose metabolism and hence glucose concentration in circulation [58]. Besides, inhaled PMs could induce an autonomic nervous system imbalance, which directly affected insulin sensitivity [59, 60]. Moreover, previous studies suggested that exposure to air pollution induces oxidative stress and adipose tissue inflammation, which disrupts insulin signaling and results in insulin resistance [61, 62]. Insulin resistance could in turn increase FBG, 1-h, and 2-h glucose concentrations. Moreover, exposure to air pollution may also affect the methylation of genes related to glucose metabolism. The change in methylation patterns affects glucose concentration by altering peripheral insulin sensitivity during pregnancy [63, 64]. Exposure to PMs could change the pancreas function and related glucose consequences. A rat model study by Yi et al. 2017 showed that exposure to PM_2.5_ has reduced pancreas glucose transporter2 (GLUT2) expression as one of the important factor of glucose intolerance as well as pancreatic methane dicarboxylic aldehyde (MDA) and suggested that inflammation and oxidative stress response related exposure to PM_2.5_ could increase risk of pancreatic impairment and glycemic consequence [65]. Other animal studies also reported that exposure to air pollution had been associated with increase in insulin immunodensity of pancreatic islets [66, 67]. Finally, changes in glucose homeostasis in healthy pregnant women might be due to the metabolic induction change in the hypothalamus [68, 69]. Our results of the associations between traffic indicators as well as PMs exposures and glucose intolerance could be explained by one or all of the above mechanisms.

The advantage of our study included the use novel markers, access to full residential address histories and detailed information on exposures. Moreover, we studied the preconception exposure to air pollution as well as traffic indicators and glucose homeostasis during pregnancy, which was not considered in previous studies. Furthermore, this study is the first report of low and middle-income countries (LMICs) about air pollution exposure and glucose intolerance in pregnant women.

Our study has limitations, as well. The sample size of our study was relatively small. We measured PMs exposure using the LUR models, and we did not measure individual exposure to PMs before pregnancy. Diet can also affect blood glucose concentrations during pregnancy, which was not assessed in our study. Furthermore, we did not evaluate the level of maternal stress that may affect blood glucose levels. These limitations should be considered in future studies.

## Conclusion

We found higher PMs exposures were significantly associated with higher risk of glucose intolerance in healthy pregnant women. Moreover, we found a significant positive association between total street length in 100 m buffer and FBG and 1-h post-load glucose concentrations. Furthermore, a significant negative association was observed between proximity to major roads and FBG and 1-h post-load glucose concentrations. Our finding provided evidence linking traffic indicators and PMs exposure with glucose homeostasis in pregnant women. If our results replicated by future studies could be a primary target in interventions to prevent glucose metabolism abnormalities. Moreover, our findings could offer evidence base for policymakers to implement interventions targeted at reducing adverse health effects of exposure to air pollution in pregnant women in our rapidly urbanizing world. However, further longitudinal studies with larger sample size are needed to confirm these results.

## Supplementary Information

**Additional file 1: Table S1.** The predictor variables and performance indicators of developed land use regression (LUR) models of annual mean PMs. **Fig. S1.** Correlation plot of FBS, PP1 and PP2 and main independent variables (i.e., PMs and traffic indicators).

## Data Availability

The data are available from the corresponding author upon reasonable request.
