# Supplementary material for: Preconception air pollution exposure and glucose tolerance in healthy pregnant women in a middle-income country
Source: Environ Health. 2020 Dec 9;19:131. doi: 10.1186/s12940-020-00682-y (PMC7727159; doi:10.1186/s12940-020-00682-y)
Supplement: Supplementary file 1 — Additional file 1: Table S1. The predictor variables and performance indicators of developed land use regression (LUR) models of annual mean PMs. Fig. S1. Correlation plot of FBS, PP1 and PP2 and main independent variables (i.e., PMs and traffic indicators). [file 12940_2020_682_MOESM1_ESM.docx]

**Supplemental Materials**

**Preconception air pollution exposure and glucose tolerance in healthy pregnant women in a middle income country**

**Moslem Lari Najafi, Mehdi Zarei, Ali Gohari, Leyla Haghighi, Hafez Heydari, Mohammad Miri**

**Table S1 …………………………………………………………….. page 2**

**Fig S1 ………………………………………………………………... page 3**

**Table S1:** The predictor variables and performance indicators of developed land use regression (LUR) models of annual mean PMs.

| **Pollutant** | **Equation** | **R^2^** | **R** | **RMSE** | **LOOCV**  **RMSE** | **Max VIF (variable)** |
| --- | --- | --- | --- | --- | --- | --- |
| PM_1_ | 33.52 + 2.12E-02* **DIST to RelCu** + 2.66E-03* **Ind_500** -1.29E-04* **Edu_500** | 0.68 | 0.82 | 5.21 | 6.18 | 6.40  (Edu_500) |
| PM_2.5_ | 41.70 + 3.28E-03* **Ind_500** + 2.30E-02* **DIST to RelCu** - 0.62***MH_200** | 0.71 | 0.95 | 3.33 | 4.7 | 3.56  (MH_200) |
| PM_10_ | 53.39 + 1.77E-03***Othr_100** + 4.05E-02* **DIST to RelCu** -4.14E-03* **DIST to BuT -** 9.52E-03* **DIST to UrFa** | 0.75 | 0.86 | 8.99 | 10.34 | 4.49  (Othr_100) |

**Note: DIST to BuT** = distance to bus terminal**; DIST to RelCu** = distance to religion/cultural land use**; DIST to UrFa =** distance to urban facility land use**; Edu** = education land use area; **Ind** = industrial land use; **MH =** maximum height of building**; Othr** = other land use area.

**
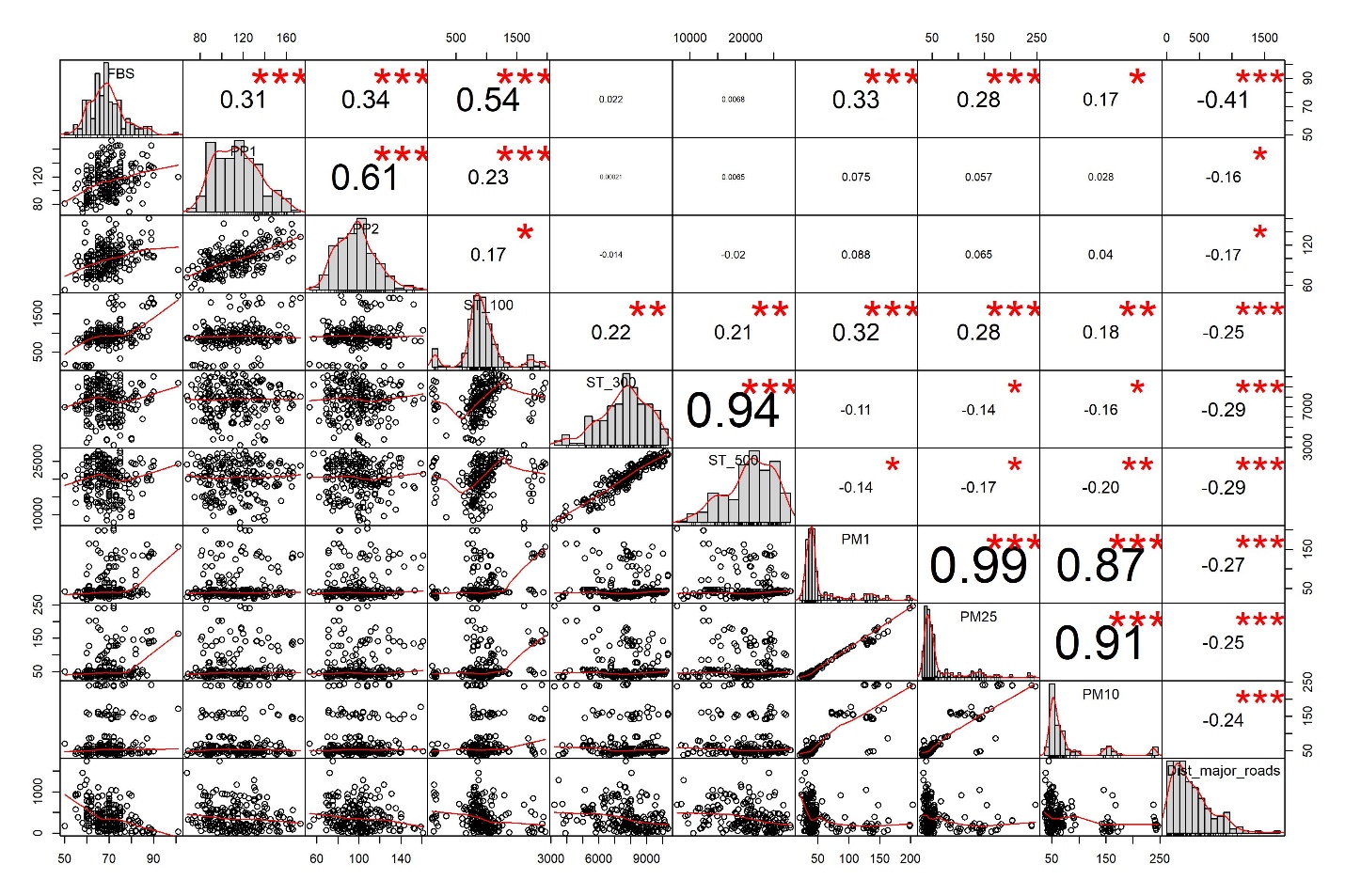
**

**Fig S1:** Correlation plot of FBS, PP1 and PP2 and main independent variables (i.e., PMs and traffic indicators)
